# The Role of Perirenal Adipose Tissue in Carcinogenesis—From Molecular Mechanism to Therapeutic Perspectives

**DOI:** 10.3390/cancers17071077

**Published:** 2025-03-23

**Authors:** Adriana Grigoraș, Cornelia Amalinei

**Affiliations:** 1 Department of Morphofunctional Sciences I, “Grigore T. Popa” University of Medicine and Pharmacy, 700115 Iasi, Romania; cornelia.amalinei@umfiasi.ro; 2 Department of Histopathology, Institute of Legal Medicine, 700455 Iasi, Romania

**Keywords:** perirenal adipose tissue, cancer, metastasis, adipokines, prognosis, predictive factor, cancer therapy

## Abstract

Perirenal adipose tissue (PRAT) exhibits particular morphological features, with its activity being mainly related to thermogenesis. However, an expanded PRAT area seems to play a significant role in cardiovascular diseases, diabetes mellitus, and chronic kidney disease pathogenesis. Numerous studies have demonstrated that PRAT may support cancer progression and invasion, mainly in obese patients. The mechanism underlying these processes is of dysregulation of PRAT’s secretion of adipokines and pro-inflammatory cytokines, such as leptin, adiponectin, chemerin, apelin, omentin-1, vistatin, nesfatin-1, and other pro-inflammatory cytokines, modulated by tumor cells. Cancer cells may also induce a metabolic reprogramming of perirenal adipocytes, leading to increased lipids and lactate transfer to the tumor microenvironment, contributing to cancer growth in a hypoxic milieu. In addition, the PRAT browning process has been specifically detected in renal cell carcinoma (RCC), being characterized by upregulated expression of brown/beige adipocytes markers (UCP1, PPAR-ɣ, c/EBPα, and PGC1α) and downregulated white fat cells markers, such as LEPTIN, SHOX2, HOXC8, and HOXC9. Considering its multifaceted role in cancer, modulation of PRAT’s role in tumor progression may open new directions for oncologic therapy improvement. Considering the increasing evidence of the relationship between PRAT and tumor cells, our review aims to provide a comprehensive analysis of the perirenal adipocytes’ impact on tumor progression and metastasis.

## Simple Summary

Perirenal adipose tissue (PRAT) is currently recognized as a significant factor involved in different cardiovascular and chronic renal diseases. However, limited data are available in published literature regarding perirenal adipose tissue’s adapted morphology and its molecular activity in cancer. PRAT releases a large spectrum of adipokines that may promote tumor cells’ growth and metastasis in several types of cancer. Cancer cells may also induce a PRAT browning process and a metabolic reprogramming to support their growth in the hypoxic tumor microenvironment. In this context, our review represents an update of the current knowledge regarding PRAT’s role in cancer development and progression and its therapeutic potential in oncologic patients.

## 1. Introduction

Classically considered a lipid storage tissue in the fat visceral compartment, perirenal adipose tissue (PRAT) also acts as an endocrine organ that produces a large spectrum of adipokines involved in the molecular mechanisms associated with diabetes mellitus, chronic kidney disease, cardiovascular diseases, and cancer [1,2].

Anatomically, the renal fascia separates PRAT from the pararenal adipose tissue of the retroperitoneal space [3]. Morphologically, PRAT is a unique type of visceral adipose tissue (VAT) consisting of a cellular complex that includes white adipocytes, brown adipocytes, ‘dormant’ brown adipocytes, adipose stem cells, endothelial cells, pericytes, a few immune cells associated with an extracellular matrix, and numerous nerve endings [4,5,6]. In addition, recent studies have revealed that adult kidney donor PRAT includes a distinct population of dormant uncoupling protein 1 (UCP1)+ adipocytes with unilocular features and gene profiles that partially overlap with those of white adipocytes [4,7]. Currently, the PRAT core is considered a specific area of visceral brown adipose tissue with high UCP1 expression, while its exterior mainly consists of adipocytes with unilocular features expressing white fat markers [8]. However, the cellular composition of PRAT changes, due to a continuous replacement of brown adipose cells by white adipose cells, in the human ageing process [8].

Although PRAT is mainly involved in thermogenesis, it seems to play multiple roles in the human body [3,9]. Recently, perirenal adipocytes have been identified as strong candidates for cancer cell growth promoters, with a consistent impact on cellular modulation of the tumor microenvironment [3]. In this regard, PRAT-derived adipokines and their adapted metabolisms have been associated with the progression of several types of cancers, such as renal, gastric, colorectal, ovarian, or prostate cancers [10,11,12,13,14]. Thus, the disturbed balance of PRAT-derived leptin and adiponectin, added to other adipokines, may support cancer cell growth in the tumor microenvironment, especially in obese patients [15,16]. Adipokine secretion dysfunction in oncologic patients is also induced by the immune cells, which infiltrate the expanded PRAT area [2,17]. PRAT also modulates vascular endothelial growth factor A (VEGFA), vascular endothelial growth factor D (VEGFD), Jagged 1 (JAG1), and transforming growth factor β1 (TGF-β1) activity in the tumor niche, promoting tumor angiogenesis and, consequently, cancer cell growth and metastasis [16,17,18].

Moreover, tumor cells are forced to adopt different strategies to enhance their nutrient intake in the hypoxic tumor niche. As a result, increased lipolysis associated with the PRAT ‘browning’ process supports the cancer cells’ high lipids requirements [19,20]. Consequently, PRAT adipocytes adopt beige/brown morphology features and exhibit overexpression of brown/beige adipocyte markers, such as UCP1, peroxisome proliferator-activated receptor ɣ (PPAR-ɣ), CCAAT/enhancer binding protein α (c/EBPα), and peroxisome proliferator-activated receptor gamma coactivator-1α (PGC1α), in cancer [20]. Furthermore, PRAT metabolic reprogramming is characterized by increased lactate secretion of beige perirenal adipocytes in the hypoxic cancer niche [19]. Considering these accumulated data, PRAT may be regarded as a visceral adipose tissue that is able to influence both cellular and non-cellular components of the tumor microenvironment, promoting cancer development and metastasis.

Another process currently studied in experimental models is that of the molecular crosstalk between PRAT and tumor cells in kidney cancer, leading to promising results regarding new therapies targeting perirenal adipocytes [19,21]. Thus, adiponectin and adiponectin receptor 1 (AdipoR1) agonists, inhibition of monocarboxylate transporter (MCTs), modulation of leptin and peroxisome proliferator-activated receptors (PPARs) expression, of chemerin-CMKLR1 pathway or hypoxia-inducible factor (HIF) inhibitors administration, added to combined therapy, including tyrosine kinase inhibitors (TKIs) and thermogenesis inhibitors, are future therapeutic tools targeting PRAT adipocytes [19,22,23,24,25,26,27]. In the context of recent scientific breakthroughs, this review aims to explore PRAT activity and the underlying molecular mechanisms associated with tumor progression in different cancers, which may provide new therapeutic perspectives in oncologic patients.

## 2. PRAT-Adapted Morphology in Cancer

There is increasing evidence that brown-like adipocyte morphological changes occur in cancer’s surrounding white fat areas [20,28]. The ‘browning’ conversion in visceral adipose tissue (VAT) may be supported by cancer cells and by the oncoinflammatory niche, which is characterized by high level of pro-inflammatory cytokines, such as interleukin 6 (IL-6) or tumor necrosis factor α (TNF-α). This enhanced inflammatory status or pro-inflammatory profile of the tumor microenvironment has been shown to contribute to increased lipolysis and cancer cachexia in variable types of cancers through *PRDM16* gene overexpression via activation of the STAT3 pathway [29,30]. In addition, visceral fat white-to-brown adipocyte conversion is generally characterized by adipocytes’ UCP1 overexpression and cellular metabolism activation [19,28]. Due to these changes, the white adipocytes adopt brown-like features, associated with metabolic adaptation in visceral fat areas, releasing energetic substrates and metabolites in the tumor microenvironment that support cancer cells’ growth [19,20,31].

Similarly, several studies have demonstrated that tumor cells have the ability to induce the conversion of the adipocytes located near the tumor invasive front into so-called cancer-associated adipocytes (CAAs), which exhibit loss of fatty acid-binding protein 4 (FABP4), a terminal adipocyte differentiation marker [32,33,34]. However, a previous study showed that RCC cells induce a beige adipocyte transformation, different from CAAs, in human adipose explants from kidney cancer (hRAT), without any alteration of FABP4 expression [19]. Taken together, these data may support the hypothesis that cancer cells promote a white-to-brown PRAT conversion, which is characterized by different features from those registered in analogous processes in subcutaneous or visceral adipose tissue.

Although several studies have reported the cancer-associated PRAT ‘browning’ process, the in-depth mechanism associated with its phenotypical changes is only partially deciphered [15,19,20,28]. In this regard, *Prdm16* and *Tbx1* genes’ overexpression, along with the increased expression of brown/beige adipocytes markers (UCP1, PPAR-ɣ, c/EBPα, and PGC1α), have been recently demonstrated in hRAT [20]. Comparable data have been reported by Wei et al., with *LEPTIN* and *SHOX2* genes’ reduced expression in the perirenal area immediately adjacent to a renal tumor, compared to those of perirenal adipocytes located 5 cm away from the tumor site [19]. Supplementary, peritumoral PRAT adipocytes display a higher expression of brown fat markers (PGC1α, DIO2, UCP1, CIDEa, and Tbx1) and an increased level of mitochondrial proteins compared to distal PRAT adipocytes [19]. Furthermore, a decreased amount of PRAT white adipocytes was also noted in another group of RCCs exhibiting UCP1 overexpression, associated with decreased expression of specific white adipocyte markers, such as homeobox C8 (HOXC8) and homeobox C9 (HOXC9) [28]. Moreover, the same study did not reveal any significant difference regarding UCP1 expression or any differences in adipocyte morphology between perirenal adipocytes located in the kidney’s lower and upper poles [28]. These findings may suggest that white adipocytes’ transdifferentiation into beige adipocytes occurs in human PRAT and that this white-to-brown transdifferentiation is more significant in PRAT areas located adjacent to RCC tumor mass. Furthermore, based on the mixed cellular composition of the perirenal fat, it has been suggested that the PRAT ‘browning’ process occurs due to the beige/brown fat cell progenitors’ activation and/or of transdifferentiation of white adipocytes into brown/beige fat cells in kidney cancer [20]. However, additional studies are needed to validate the contribution of these alternative mechanisms to the PRAT ‘browning’ process. The relationship between perirenal adipocytes’ UCP1 overexpression and the morphological changes that may occur during carcinogenesis seems to be associated with RCC inner-type growth, suggesting a possible role of PRAT browning prevention of outward RCC growth [15]. Not least, the activation of PRAT latent brown adipocytes, which has been recently identified in PRAT deposits of adult kidney donors, may potentially be involved in tumor progression [4,35].

## 3. Potential Mechanisms of Tumor Cell–PRAT Interrelationship

### 3.1. PRAT-Derived Adipokines in Cancer

Obesity, a condition associated with a body mass index (BMI) over 30 kg/m^2^, is currently considered a pivotal risk factor in a large spectrum of pathologies, such as cardiovascular and renal chronic diseases, and diabetes mellitus [3,9]. In addition, numerous studies have demonstrated that excess visceral fat plays a significant role in carcinogenesis [2,31,36]. Moreover, as a part of visceral adipose tissue, PRAT’s involvement in cancer development and its metastasis has been recently demonstrated in obese patients, mainly in clear cell renal cell carcinoma (ccRCC), as well as ovarian and colorectal cancer [37,38,39].

PRAT increase induces dysfunction of adipokine secretion and local chronic inflammation, modulating most of the obesity-related tumors through paracrine or endocrine pathways [2,16,17]. A large panel of adipokines, including leptin, adipokine, chemerin, apelin, omentin-1, vistatin, nesfatin-1, and other pro-inflammatory cytokines (IL-6 and TNF-β) produced by excessive PRAT may contribute to tumor progression by inducing a pro-inflammatory, dysmetabolic, and pro-angiogenic tumor microenvironment [3,16,17,40,41]. Consequently, the peak of these events leads to a cascade of effects, such as immune clearance mechanism disruption, cancer cell metabolism reprogramming, and tumor invasion and metastasis facilitation [2,17].

**Figure 1:**
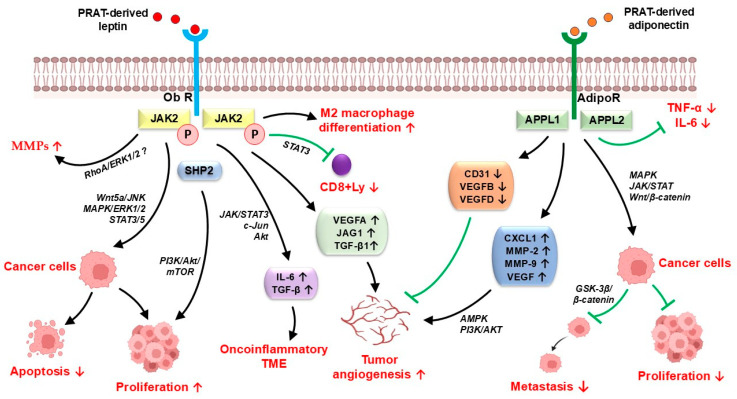
Potential mechanisms of PRAT-derived leptin and adiponectin in cancer progression and metastasis. PRAT-derived leptin binds to its receptor (Ob-R), promoting tumor growth and metastasis by JAK/STAT3, PI3K/Akt/mTOR, Wnt5a/JNK, and MAPK/ERK1/2 pathways, modulates the anti-tumor immune response by M2 macrophage differentiation, and is involved in extracellular matrix degradation via RhoA/ERK1/2 signaling stimulation. PRAT-derived adiponectin binds to its receptor (AdipoR), leading to the recruitment of APPL1 and APPL2, followed by the activation of several signaling pathways associated with anti-tumor effects and reduction of the tumor microenvironment inflammatory features by decreased expression of TNF-α and IL-6, but it also promotes tumor angiogenesis via AMPK and PI3K/AKT pathway activation. Adipo-R—adiponectin receptor; CD31—cluster differentiation 31; CXCL1—chemokine (C-X-C motif) ligand 1; IL-6—Interleukin 6; MMP—matrix metalloproteinase; Ob-R—leptin receptor; PRAT—perirenal adipose tissue; TME—tumor microenvironment; TNF-α—tumor necrosis factor α; VEGFA—vascular endothelial growth factor A; VEGFB—vascular endothelial growth factor B; ↑—increase; ↓—decrease; →—activation; ꓕ—inhibition.

Leptin, mainly released by white adipocytes, has been demonstrated to act as a significant cancer-associated adipokine in different types of malignancies, including colorectal, breast, liver, and ovarian cancer, and malignant melanoma [42,43]. Leptin promotes cancer cell progression and metastasis by tumor cell proliferation induction and cancer cell apoptosis inhibition via Wnt5a/JNK, STAT3/5 and MAPK/ERK1/2 pathways activation (Figure 1) [2,44]. In addition, leptin may induce tumor cell growth through PI3K/Akt/mTOR pathway stimulation [45]. High leptin expression has also been associated with poor prognosis and invasion of tumor cells by matrix metalloproteinases (MMPs) overexpression that induces the degradation of the extracellular matrix components [46]. Leptin also promotes M2 macrophage differentiation in the tumor microenvironment, a process associated with tumor progression and lymph node metastasis [33,47]. Leptin also acts as a pro-inflammatory adipokine that induces IL-6 and transforming growth factor β (TGF-β) overexpression and an oncoinflammatory tumor microenvironment development via JAK/STAT3, c-Jun, and Akt pathway activation [33,48]. These findings are supported by another study that demonstrated a decreased tumor cells migration by IL-6R activation blockage with IL-6 antibody or with inhibitors of the STAT3/JAK pathway [10].

Although incompletely studied, PRAT-derived leptin imbalance in the tumor microenvironment has been recently noticed in several studies [15,16]. In this respect, leptin overexpression in PRAT adipocytes has been identified in RCC patients [16]. According to the same study results, PRAT area expansion has been associated with *VEGFA*, *JAG1*, and *TGF-β1* pro-angiogenesis gene expression increases, suggesting a possible interplay between PRAT-derived leptin activity and tumor angiogenesis [16]. This finding may contribute to a new targeted therapy development in RCC patients, based on anti-VEGF oncotherapy response and, consequently, to the patient’s prognosis improvement [16]. In contrast, in another study, the analysis of leptin expression in perirenal adipocytes and human RCC growth pattern did not detect any significant correlations [15]. Due to these contradictory findings, future research may explore the underlying mechanism of PRAT-derived leptin involvement in tumor development and progression. Moreover, according to our knowledge, there are no published data regarding the role of PRAT-derived leptin in other types of cancers, necessitating supplementary studies in the near future.

The pro-oncogenic leptin activity is thought to be counterbalanced by adiponectin, another significant adipokine released by perirenal adipocytes [15,17]. Adiponectin exerts anti-tumor activity by suppressing the secretion of pro-inflammatory cytokines, e.g., IL-6 and TNF-α, lessening the chronic inflammation related to obesity or the anti-inflammatory effect [49]. In addition, adiponectin may induce apoptosis and downregulates survival and proliferation of tumor cells through MAPK, JAK/STAT and Wnt/β-catenin pathway activation [2]. Accumulating data have also indicated that adiponectin may act as an anti-angiogenic factor in the tumor microenvironment by the decreased expression of CD31, VEGFB, and VEGFD and interleukin-12 (IL-12) activity modulation (Figure 1) [18,50,51].

Moreover, epidemiological evidence has revealed a negative correlation between the adiponectin serum level and the risk of obesity-related cancers [2,52]. In this regard, a decreased adiponectin serum level has been demonstrated to be associated with tumor cell growth in RCC patients [15,53]. Another recent study revealed that PRAT thicknesses is associated with downregulation of adiponectin expression in perirenal adipocytes in a large group of metastatic RCC patients [16]. A reduced adiponectin serum level has also been identified in patients with metastatic kidney cancer, while a high preoperative adiponectin serum level has been associated with an 83% decrease in risk of death in RCC patients [17,54]. In addition, a negative correlation between adiponectin plasma level and tumor size and metastasis has been registered in RCC [55]. Another in vitro study demonstrated AdipoR1 downregulation in ACHN and Caki-1 renal tumor cell lines, following their incubation with hRAT in conditioned media, by comparison with normal human adipose explants [56]. Moreover, AdipoR1 activation may reduce tumor cell migration by GSK-3β/β-catenin pathway blockage in RCC [11]. A decline of the adiponectin plasma level may be also attributed to overexpression of TNF-α in the expanded PRAT area of obese patients [57,58]. This pro-inflammatory cytokine modulates insulin sensitivity and, in consequence, PRAT adiponectin secretion [57,58].

Nonetheless, a recent study has been shown no significant difference between *LEP* and *ADIPQ* gene expression in a group of RCC patients compared to a control group [28], while other studies find no significant differences in adiponectin expression of human adipose explants by comparison with normal (hRAN) or kidney cancer (hRAT) cells in conditioned media [20,59]. Moreover, no correlations between tumor growth pattern and leptin or adiponectin immunoexpression of perirenal adipocytes [15] or between serum adiponectin level and BMI or RCC survival have been identified in other study groups of RCC patients [60]. Taken together, these preliminary data regarding the adiponectin impact on carcinogenesis should be explored in future clinical studies, in the context of the reports of cancer promotion and tumor cells invasion effects, supported by newly developed vessel density or pro-angiogenic adiponectin activity in tumor microenvironment [61,62,63]. In this respect, adiponectin may stimulate angiogenesis by inducing proliferation and migration of the endothelium via AMPK and PI3K/AKT pathway activation [18]. AdipoR1 activation may also enhance the expression of different pro-angiogenic factors, such as chemokine (C-X-C motif) ligand 1 (CXCL1), MMP-2, MMP-9, and VEGF, that induce tumor blood vessel formation [64,65]. Considering these accumulated data, the adiponectin effects in tumor angiogenesis remain only partially deciphered. In this regard, it has been suggested that the pro- or anti-angiogenic adiponectin role is modulated by the cancer cells’ ability to create a specific tumor microenvironment that supports their growth and invasion. The study results’ discrepancies may be also partially attributed to the cellular and molecular complexity of the tumor microenvironment, considering that the balance between PRAT-derived leptin and adiponectin level may be a key factor that promotes carcinogenesis. Nonetheless, a better understanding of the balance between PRAT-derived leptin and adiponectin in the tumor microenvironment may open new therapeutic perspectives in this category of oncologic patients.

**Figure 2:**
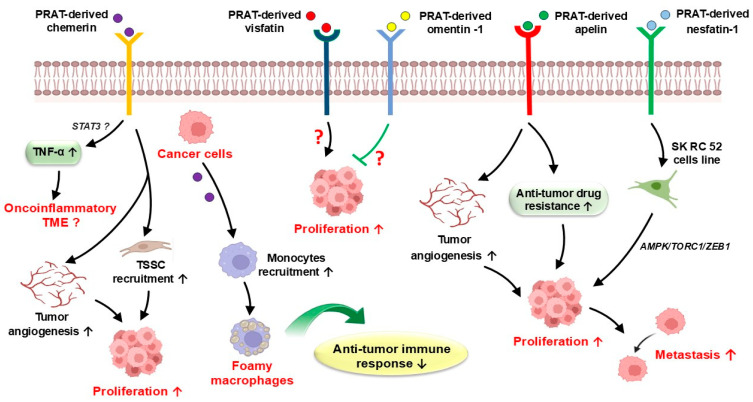
Potential mechanisms of PRAT-derived chemerin, visfatin, omentin-1, apelin and nesfatin-1 in cancer progression and metastasis. PRAT-derived chemerin promotes tumor angiogenesis, tumor-supporting stromal cell recruitment, contributes to the tumor’s pro-inflammatory features by TNF-α expression increase via STAT3 pathway activation, and may modulate the anti-tumor immune response by monocyte recruitment and their change into foamy macrophages. PRAT-derived visfatin, omentin-1, and apelin support cancer cell proliferation, while PRAT-derived nesfatin-1 may promote tumor cell metastasis by AMPK/TORC1/ZEB1 pathway activation. PRAT—perirenal adipose tissue; TNF-α—tumor necrosis factor α; TSSC—tumor-supporting stromal cells; ↑—increase; ↓—decrease; →—activation; ꓕ—inhibition.

Limited literature data are available regarding the potential role of other PRAT-derived adipokines (e.g., chemerin, visfatin, omentin-1, and apelin) in kidney pathology and tumor biology [58,66]. Nevertheless, recent studies demonstrated that chemerin is a significant pro-inflammatory cytokine produced by fat cells or cancer cells, which displays a pro-tumoral activity by stimulation of the recruitment of tumor-supporting stromal cells and by activation of angiogenesis signaling in the tumor microenvironment [35,66,67]. An increased expression of tumor cell-derived chemerin has been also suggested to be associated with monocyte recruitment and their transformation into foamy macrophages, due to lipid accumulation in their cytoplasm, in the RCC tumor microenvironment (Figure 2) [68].

These data support the potential relationship between chemerin and anti-tumor immune response [2,69]. However, a recent study has reported that higher chemerin levels are not correlated with increased chemerin bioactivity [70], even though increased chemerin secretion may be induced by TNF-α, a pro-inflammatory cytokine, which is highly expressed by PRAT in obesity [71]. Therefore, further research is needed to explore the precise molecular mechanism and the multifaceted role of PRAT-derived chemerin in tumor development, considering that chemerin inhibitors have already been proposed as potential modulators of the anti-tumor immune response [21].

Visfatin has been characterized as an adipokine that modulates cancer cell viability [72]. Although different studies have revealed that visfatin overexpression and increased visfatin serum level are associated with higher Fuhrman grades and poor survival outcome in RCC patients, its potential role in carcinogenesis remains to be demonstrated [41,73,74].

Omentin-1 is another adipokine, mainly released by visceral adipose tissue, including PRAT [41]. A negative association between omentin-1 serum level and cancer-associated obesity has been demonstrated, suggesting the potential role of omentin-1 as a tumor progression indicator [2,75]. Additionally, omentin-1 may act as a tumor-suppressor factor, as a lower omentin-1 serum level has been reported in RCC patients [41].

Apelin is related to ovarian, renal, and breast carcinogenesis and cancer progression [18]. Apelin expression has been detected in different organs and tissues, including the lung, pancreas, prostate, brain, heart, placenta, and adipose tissue [76]. Currently, it is considered that apelin supports tumor cell growth and metastasis by enhancing angiogenesis and anti-tumor drug resistance development [41]. However, insufficient literature data regarding PRAT-derived apelin in carcinogenesis are available. Moreover, some studies that explored the association of apelin with RCC have revealed inconsistent and contradictory results, most of them demonstrating no association between apelin gene expression and overall survival in RCC patients [18,41].

Limited literature data regarding nesfatin-1’s potential role in RCC progression are currently available [77,78,79]. However, inhibition of tumor nodule formation has been associated with nesfatin-1 upregulation in a RCC murine model [41]. A direct correlation between Fuhrman grades and overexpression of nesfatin-1 was also reported in SK RC 52 cells line (derived from ccRCC metastatic mediastinum lesions), suggesting its potential involvement in RCC progression by AMPK/TORC1/ZEB1 pathway activation (Figure 2) [79]. Additionally, a strong association between nesfatin-1 expression and high-grade RCC (OR = 0.29, 95% CI = 0.13–0.61) has been demonstrated, suggesting the predictor value of nesfatin-1 for RCC aggressiveness [41].

Considering this accumulated information, PRAT-derived adipokines may play a significant role in tumor cell growth and progression. However, the interplay between PRAT-derived adipokines and cancer is still only partly understood. As a result, future research is required to confirm the possible role of some PRAT-derived adipokines, such as apelin, omentin-1, and nesfatin-1, in cancer cell growth and tumor invasion.

### 3.2. PRAT-Induced Hypoxia and Inflammation in Cancer

Visceral adipose tissue expansion has been shown to induce local hypoxia, which may act as a trigger for a compensatory angiogenesis mechanism [80]. In addition, enlargement of the adipose area is associated with a lower oxygenation value of the adipose tissue in obese people compared to lean patients (39.3 ± 1.5 mm Hg vs. 53 ± 1.9, *p* < 0.001) [81]. The hypoxic obese PRAT microenvironment may promote cancer progression by hypoxia-induced factor 1α (HIF-1α) signaling activation and upregulation of CCL2, TNF-α, IL-10, IL-8, and IL-6 pro-inflammatory cytokine expression via NF-κB-pathway stimulation [80,82]. The local hypoxic state is also associated with an increased PRAT infiltration with immune cells in obese patients, which may also lead to a pro-inflammatory tumor microenvironment that promotes angiogenesis and tumor metastasis [3,35,80].

However, it has been suggested that HIF-1α modulates tumor cell survival by blocking the pro-apoptotic axis, a reduction in tumor size being related to HIF-1α overexpression [83]. Additionally, it may lead to Myc complexing in an inactive form, which is related to cell cycle arrest [83].

Available data show that HIF-1α activation may also inhibit the effects of toxic reactive oxygen species (ROS) on tumor cells and may modulate glycolysis and pyruvate metabolism, as well as cancer cell metastatic potential and resistance to oncotherapy [84]. HIF-1α may support ccRCC initiation by expression of genes involved in Warburg metabolism, with increased rates of conversion of glucose to lactate and reduced rates of pyruvate entry into mitochondrial respiration [85]. The carbohydrate metabolism alteration related to HIF-1α is associated with glycogen accumulation and failure to efficiently metabolize fatty acids [85]. In turn, HIF-2α-related hypoxia’s pro-tumorigenic role has been demonstrated in RCC [80]. In this regard, it has been shown that overexpression of HIF-2α may support renal cancer cell proliferation [85]. Moreover, HIF-2α modulates the activity of genes that regulate cholesterol and lipoprotein uptake and metabolism, leading to the occurrence of the clear cell phenotype [85].

**Figure 3:**
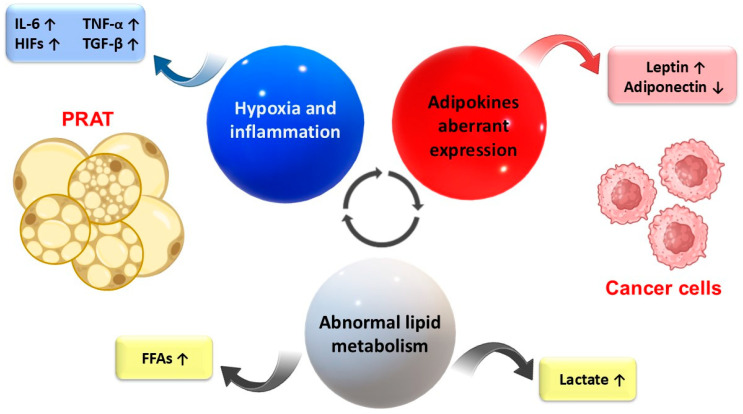
Potential mechanisms linking cancer to PRAT dysfunction in the tumor microenvironment. Aberrant adipokine expression and abnormal lipid metabolism, along with the increased hypoxic and inflammatory features of the tumor microenvironment, are the main mechanisms of tumor cell–PRAT interrelationship. FAAs—free fatty acids; HIFs—hypoxia-inducible factors; IL-6—Interleukin 6; PRAT—perirenal adipose tissue; TGF-β—transforming growth factor-β; TNF-α—tumor necrosis factor α; ↑—increase; ↓—decrease.

Such findings underline the hypothesis that HIF-1α may exhibit tumor suppressor activity, while HIF-2α supports tumor progression [80]. However, HIF-1α may also induce VEGF overexpression, leading to tumor neoangiogenesis [86]. Furthermore, overexpression of HIF-1α is associated with a worse prognosis in CRC patients [87,88]. Therefore, despite some potential beneficial effects of HIF-1α, overall, both HIF-1α and HIF-2α are currently thought to be involved in different stages of tumor development, their activity being modulated by numerous mutations, which are associated with tumor progression (Figure 3).

Visceral fat expansion, including that of PRAT, has been associated with local pro-inflammatory features related to changes of immune cellular composition and their inflammatory cytokine imbalance [15,17,80]. In this regard, inflamed visceral adipose tissue becomes a source of TNF-α, which induces pro-angiogenic factor overexpression as well as upregulation of anti-apoptotic factors, such as cyclin D1, cyclin E, and Bcl-2, that contribute to cancer cells’ survival and migration [80,89]. Additionally, Th1 lymphocyte activation may lead to the increased secretion of IFN-ɣ, which supports local adipose tissue inflammation [80]. Apart from these changes, perirenal adipocytes are able to support the local pro-inflammatory status through increased secretion of other cytokines, e.g., MCP-1 and IL-6 [17,80]. These findings are reinforced by a recent study, demonstrating that the PRAT expanded area releases high amounts of IL-6, TGF-β, and TNF that support the RCC cells’ progression by overexpression of genes related to angiogenesis [16]. IL-6, IL-10, and HSP-90 PRAT expression may be also used as potential markers of RCC progression [80].

In addition to higher leukocyte and neutrophil counts, an increase of macrophages infiltration occurs in visceral fat areas in obese patients through CCL2/IL-1β/ CXCL12 pathway activation [90,91]. The macrophages’ recruitment and higher expression of IFN-ɣ induce the transformation of macrophages in the inflammatory (M1) phenotype via cJun NH2-terminal kinase pathway activation [92]. Leptin may also promote M2 macrophage activation and their secretion of pro-inflammatory cytokines that facilitate tumor progression and metastasis [18]. The activated macrophages induce tumor progression through stimulation of angiogenesis and secretion of a large panel of pro-inflammatory cytokines, including cyclooxygenase-2 (COX-2), IL-6, plasminogen activator inhibitor-1 (PAI-1), and TNF-α, that contribute to an oncoinflammatory tumor microenvironment development [80]. In this respect, an increased COX-2 PRAT expression has been detected in 153 consecutive cT1 RCC patients who underwent nephrectomy, corresponding to an inflammatory status of the perirenal fat area in obese patients [15]. Therefore, the adipocytes regulate the immune cell infiltration and macrophage activation, leading to an immunosuppressive microenvironment and, consequently, to tumor cells’ immune escape.

Moreover, the pro-inflammatory adipokines support tumor angiogenesis and extracellular matrix remodeling by modulation of other non-tumor associated cells’ (e.g., tumor-associated fibroblasts and endothelial cells) activity [2]. As a result, PRAT adipocyte activity contributes to a pro-inflammatory niche development that promotes cancer cell growth and may have an impact on tumor progression in obesity. However, future research is warranted to characterize the PRAT inflammatory profile in obesity-related cancer, considering its complex cellular composition and the particular brown-like phenotype that may lead to functional heterogeneity.

### 3.3. PRAT-Derived Metabolic Dysfunctions in Cancer

Tumor cells exhibit different metabolic adaptations that allow them to accumulate nutrients, such as cholesterol, glucose, glutamine, and amino acids, to sustain their survival in a hostile tumor microenvironment [93]. Consistent research evidence has shown that increased endogenous lipid synthesis and exogenous lipid uptake support tumor cells’ membrane synthesis and modulation of their lipid-mediated pathways, stimulating cancer growth and metastasis [94,95].

Cancer cells can also actively induce metabolic changes in other non-tumor cells, such as adipocytes, which are characterized by increased lipolysis, leading to increased fat utilization by cancer cell mitochondria, as demonstrated in malignant melanoma, as well as breast and ovarian cancers [43,93]. Due to increased lipolysis, PRAT adipocytes become smaller, adopt multilocular features, and contribute to a hypermetabolic status of the tumor microenvironment [20]. In this regard, growing evidence shows that cancer cell-induced metabolic adaptation of visceral adipocytes, including PRAT, is associated with increase of lipid droplets triglycerides (TGs) hydrolysis with free fatty acids (FFAs) release into the tumor microenvironment [19,96,97]. Exogenous FAAs released from perirenal adipocytes may be taken up by tumor cells and degraded to give rise to ATP required for cancer cell survival and metastatic spread (Figure 3) [19,20,82]. This process involves overexpression of FABPs and CD36, key proteins that facilitate perirenal beige-derived FFA uptake by cancer cells, followed by energy synthesis through the fatty acid oxidation (FAO) metabolic pathway activation [19,80,98]. However, colorectal and renal tumor cells preferentially use FABP5 and FABP4, as an alternative to CD36, to scavenge extracellular FFAs, unlike ovarian and prostate cancers [34]. Additionally, CD36 deletion has been associated with limited effects on the proliferation of the human ccRCC cell line 786-O treated with beige adipocytes [19]. However, the same study revealed decreased expression of the *FAO* gene in renal tumor cells, which demonstrates that FFAs are mainly transformed in neutral triglycerides stored in the tumor cells’ cytoplasm [19]. Adipocyte-derived leptin also inhibits glycolysis in CD8+ lymphocytes by activation of the enzymes involved in FAO via the STAT3 pathway, impairing their anti-tumor activity [95]. These data support the hypothesis that renal cancer cells use other PRAT derived-metabolites as the main energetic source for their growth. Moreover, some morphological types of RCC, such as ccRCC, are prone to lipid storage rather than lipid catabolism.

In addition, perirenal adipocytes’ increased expression of PPARɣ, a master regulator of BAT adipogenesis, has been detected in an experimental study [20], most probably supported by PRDM16 activation, leading to perirenal beige adipocytes’ occurrence in RCC [99]. Different studies have identified PPARɣ as a significant regulator of cancer cells’ and immune cells’ lipid metabolism [99,100,101]. In this regard, PPARɣ induces FAO activation and overexpression of genes involved in lipid metabolism in cancer cells, such as pyruvate dehydrogenase kinase 4 (PDK4), carnitine palmitoyltransferase 1 (CPT1), and fatty acid binding proteins (FABPs) [102], supporting the tumor cells’ adaptation to a hypoxic and low nutrient tumor niche. Moreover, PPARɣ promotes lipolysis in adipose areas or CAAs, by increased activity of monoglycerol lipase, adipose triglyceride lipase, and heat-sensitive lipase, contributing to tumor cell progression and metastasis [34]. Last but not least, PPARɣ controls macrophage activation, DCs differentiation, and T cell proliferation by modulation of their cellular lipid metabolism in cancer [99,102]. However, the roles of PPARɣ in tumor cells development and progression is only partially understood, considering that it may have dual tumorigenic or antitumoral effects in some cancer types [99,102]. In this respect, it has been shown that PPARɣ inhibits SIX2 activity and induces tumor cell apoptosis or may promote tumor cell development in renal cancer [103,104]. In this context, additional studies are required to elucidate the intricate interplay between PPARɣ, tumor cells, and lipid metabolism, as a step into the development of novel therapeutic tools targeting PPARs in oncologic patients.

The hypoxic tumor microenvironment is a significant trigger for HIF-1α overexpression, which regulates the hypoxic response in the tumor microenvironment.

Recent data have shown that HIF-1α may increase the expression of genes related to tumor neoangiogenesis, cancer cell proliferation, and metastasis [86,105,106]. In this respect, HIF-1α may induce the increased expression of VEGF, glycolytic enzymes, IGF2, and Bcl-2 genes, which facilitate the tumor cells’ adaptation to the hypoxic tumor microenvironment, by stem cells’ survival [106].

Hypoxia may lead to other changes of the adipocyte metabolism that could provide energetic support to neighboring cells, including cancer cells [106,107]. In this regard, HIF-1α may activate lipolysis in perirenal adipocytes in the hypoxic microenvironment, HIF-1α decreased expression being demonstrated to be associated with a limited access of cancer cells to adipose-derived lipids [80,107]. HIF-1α and HIF-2α overexpression may also induce downregulation of CPT1A in renal tumor cells, promoting FAAs’ transport to mitochondria and their cytoplasmic storage [108].

In addition to FAAs, other oncometabolites, e.g., lactate, are added to abundant adipocyte-derived FFAs in the tumor environment. Furthermore, an increased lactate secretion of beige adipocytes into the tumor milieu has been recently added to the ‘browning’ transformation of the perirenal area [19]. Moreover, numerous studies have shown a direct correlation between lactate levels in the tumor microenvironment and angiogenesis, immune tolerance, resistance to oncotherapy, and cancer growth and metastasis [107,109,110,111]. Thus, recent research has demonstrated that beige adipocytes are able to release more lactate in the tumor microenvironment compared to white perirenal adipocytes, through activation of the expression of the *LDHA* gene that controls the lactate synthesis from pyruvate [19,112]. Moreover, upregulation of the expression of *MCT1, MCT4*, and *LDHB* genes, which are associated with lactate transport and catabolism, has been identified in the human 786-O and RCC10 RCC cell lines [19]. Although significant steps have been made in elucidation of the metabolic profile of the perirenal adipocytes in the tumor microenvironment, future clinical studies are required to confirm these preclinical results.

## 4. PRAT’s Potential Value in RCC Prognosis

PRAT may contribute to RCC progression, which is considered a risk factor for ccRCC pathological T-stage stratification and cancer prognosis [113,114]. In addition, perirenal and/or renal sinus fat infiltration, but not beyond the Gerota fascia, is a criterion for RCC classification as the pT3a stage, according to the last edition of WHO [115,116]. Although fat renal invasion has been reported in 5.1–18.5% of RCCs [117,118], its prognostic value is still controversial, which is partially related to the variability of the fat compartment’s involvement. In this respect, several studies have revealed that tumor extension in the renal fat did not influence patients’ survival [115,119]. Moreover, the extension of cancer cells into multiple sites of extra-renal fat has been associated with worse outcomes than a single site tumor invasion in pT3a RCC patients [115,119]. Thus, PRAT and perisinus fat areas invasion has been associated with a poor prognosis by comparison with invasion limited to the perisinus fat area in pT3aN0M0 RCCs [119,120,121]. Moreover, another study has shown that patients with tumoral invasion of the renal sinus adipose area show a 1.63-fold higher risk of death than patients who have only PRAT invasion [122]. These observations are contradictory to other previous studies, which reported that invasion to different sites is not an independent negative prognostic factor in pT3a RCCs [123,124]. Additionally, recent research revealed that a larger perirenal fat area may display a protective role in RCC prognosis, highlighting the ‘obesity paradox’ in cancer [31]. The preventive role of a large PRAT area may be the result of a reduction of perirenal fat area density, added to decreased metabolic activity and less PRAT-derived adipokine secretion [31].

PRAT thickness has also been considered an independent risk factor of cancer progression, as it is associated with reduced progression-free survival in localized ccRCCs [113], postoperative complications [31,125], and increased risk of death [17]. In this respect, recent research has demonstrated that PRAT is an independent prognostic risk factor for surgical treatment in 342 consecutive ccRCC patients [36]. According to the same study, PRAT area extension was significantly associated with clinical manifestation, and overall survival, along with tumor size and grade. However, no statistically significant correlations have been detected between overall survival and waist circumference, BMI, and percentage of visceral adipose tissue [36]. Similarly, a significant association between progression-free survival and CT-evaluated PRAT area thickness has been reported in a cohort of 358 metastatic RCC patients treated with anti-VEGF therapy [16]. These data were completed by another previous study, which showed that PRAT area tumor invasion was associated with RCC tumor diameter, especially in the pT3a stage [126]. These observations are contradictory to those of Margulis et al., who reported that extrarenal tumor extension in perirenal or sinus fat was not an independent predictive factor for the post-surgical outcome in pT3a RCCs [123]. These controversial data may be attributed to the heterogeneity of the study groups or to other risk factors (e.g., metastases and lymph node invasion). However, these accumulated data may provide clinicians with a critical reference point for an improved stratification of the surgical prognosis of CRC patients.

Additionally, Mayo Adhesive Probability (MAP) score may estimate RCC surgery difficulty during partial nephrectomy, due to the possible adherent PRAT [17,125]. In this regard, a previous study revealed a higher MAP score and an enlarged perinephric fat area in RCC patients compared to those registered in the benign tumor study group [127], supporting the value of the preoperative evaluation of renal fat thickness as a non-invasive tool, that useful in prediction of the malignant histology of a renal tumor mass and the associated postoperative risks of partial nephrectomy [128]. Analogous results have been reported by another prospective study, reporting high MAP scores associated with older age, male gender, shorter progression-free survival, higher BMI, and larger tumor size in RCC patients [129]. Nevertheless, the study by Bernstein et al. demonstrated the lack of association between MAP score or PRAT thickness and Fuhrman grade, in a group of 317 RCC patients [130]. These findings highlight PRAT’s complex role in RCC risk stratification, even though the effect of an expanded PRAT on survival outcomes is still controversial. However, another study attempted to define another future direction associated with ccRCC pathological grade estimation by applying a deep-learning algorithm to PRAT automatic segmentation and extraction of its radiomics features on CT scan images [131]. Although, these preliminary results confirmed PRAT’s complex role in RCC stratification and prognosis, further studies on larger sample sizes are needed to validate the clinical applicability of this method [131].

## 5. PRAT’s Potential Value in Gastric and Colorectal Cancer Prognosis

Only a few studies have focused on the interaction between PRAT and gastric cancer cells [13,38,132]. However, a recent study has demonstrated that a 10.7 mm preoperative PRAT volume may represent a pivotal predictive factor of complications after laparoscopic distal gastrectomy in a large group of cT1N0, cT2N0, and cT1N1 gastric cancer patients [38]. These data support the hypothesis that PRAT volume area as measured by CT imaging may be a meaningful tool for the evaluation of postoperative risk and, additionally, for visceral fat volume assessment in these patients. Other preliminary data showed that PRAT area expansion is also an independent risk factor in long-term and short-term post-curative surgery outcomes of gastric cancer patients [13]. In support of this observation, physical exercise in patients with high PRAT volume and nutritional support in patients with low PRAT volume may improve their surgical outcomes [13].

Furthermore, gastric cancer exhibits a metastatic propensity for the omentum or peritoneum, as adipocyte-rich sites [132,133]. Thus, CT assessment of visceral fat can be used to predict the occurrence of occult peritoneal metastases in these patients [132]. Additionally, another study reported the detection of tumor emboli in PRAT lymphatic vessels in the case of malignant ascites [134]. Regarding the mechanism underlying this type of invasion, it is considered that exogenous FAAs released by adipocytes-rich areas contribute to gastric tumor cell invasion through C/EBPβ-dependent diacylglycerol acyltransferase 2 (*DGAT2*) gene overexpression [135]. Therefore, the preoperative determination of PRAT volume may constitute a risk stratification tool and may assist the formation of therapeutic decisions in gastric cancer patients.

PRAT dysfunction in obese patients seems also to be involved in colorectal cancer (CRC) progression and prognosis [14,136]. In addition to gastric cancer, PRAT thickness larger than 25.1 mm has been also demonstrated as an indicator of post-surgery complication occurrence and poor prognosis in CRC patients [14]. Additionally, PRAT surface area of ≥40 cm^2^ has been associated with more frequent surgical complications than CRC with PRAT area of <40 cm^2^, indicating that PRAT area evaluation may be a practical tool in the assessment of preoperative risk in CRC patients [137]. Moreover, no significant correlation between post-intervention complications and BMI was identified by Sönmez et al., even though BMI may be used as a predictive factor of PRAT volume [14,138]. However, Eckberg et al. reported no significant relationship between PRAT volume and overall survival after surgical intervention in CRC patients [139]. These data are supported by another study which did not detect any relationship between retroperitoneal fat thickness and higher T and N stages in 83 cases of non-metastatic CRCs [140].

All these contradictory findings may be interpreted in the context of the variability of evaluation techniques and of the limited number of studies that reported a cut-off value for PRAT volume in CRCs. However, these preliminary results may be used to identify high-risk CRC patients, who may develop post-surgical complications, enabling the modulation of therapeutic management to be targeted on these selected cases.

## 6. PRAT’s Potential Value in Prognosis of Other Cancers

Although an association between obesity and colorectal or breast cancers has been currently demonstrated, the interplay between obesity and lung cancer remains a topic of ongoing studies [141]. In this regard, it has been demonstrated that high BMI has a protective effect on lung cancer progression and confers a survival advantage in a large group of non-small cell lung cancer patients requiring pneumonectomy [142]. This contradictory association between obesity and lung cancer is termed the ‘obesity paradox’ [143]. While prior retrospective studies have generated this paradoxical association, recent studies have recorded a negative association between visceral obesity and the occurrence of different types of malignant tumors, including lung cancer [144,145,146]. However, the dysmetabolic visceral adipose tissue may support lung cancer progression and may also influence the immune antitumor response in obese patients.

Nevertheless, as a part of the visceral adipose mass, PRAT’s role in lung cancer progression is insufficiently supported by literature findings. In this regard, limited studies have reported a solid (nodular) or diffuse pattern of lung carcinoma metastasis in the PRAT area [147,148,149,150]. The perirenal lymphatic system and its association with mediastinal lymph nodes or para-aortic lymph nodes have been considered to represent lung cancer metastatic spread pathways to PRAT [147,150].

In addition, another recent study presented a rare case of infiltration of the kidney parenchyma and PRAT by metastatic lung squamous cell carcinoma (CK7-, CK20-, 34BE12+, p63+, and p40+) [151], suggesting the potential role of adipocyte-derived factors and other components of the tumor microenvironment in lung cancer metastasis to this specific area.

Other reports have described exceptional metastases of malignant melanoma [152] or prostate cancer in PRAT or perirenal fascia [12,153]. In these cases, the lymphatic and hematogenous metastatic pathways, added to the perirenal fat pro-inflammatory status in obese patients, have been suggested to support the tumor cells’ infiltration of PRAT areas.

There are still limited studies that have explored PRAT area involvement in ovarian tumor cell progression and metastasis [3]. In this respect, IL-6 derived from PRAT stromal cells has been found to be involved in ovarian cancer cell progression by JAK2/STAT3 pathway activation [10]. Additionally, adiponectin–leptin balance disturbance contributes to a pro-inflammatory status in visceral fat areas due to overexpression of IL-6 that may lead to survival of dormant ovarian tumor cells [154,155]. Moreover, IL-10 upregulation in the PRAT area may be associated with ovarian cancer growth, while inhibition of IL-12 has been related to a favorable prognosis in malignant ascites by increased IFN activity [3,37].

The PRAT ‘browning’ process has also been associated with poor prognosis in ovarian cancer, UCP1 overexpression in perirenal fat being considered as a promoter of ovarian cancer cell progression [37]. Moreover, the white-to-brown transdifferentiation of perirenal fat may lead to tumor cachexia through increased resting energy expenditure in brown adipocytes [37]. PRAT also acts as a prognosis factor in ovarian cancer, with PRAT area thickness of more than 5 mm being related to a 5 years’ lower survival in a group of patients with stage III and IV ovarian cancer [37]. Considering these data, PRAT is not only involved in tumor cell progression, but its large thickness is considered a valuable prognosis factor in ovarian cancer patients.

In light of these findings, PRAT assessment using imaging technology is now considered an additional factor for prognosis prediction in different types of cancer, not only in renal cancer. However, future in-depth research should explore PRAT-adapted morphology and the molecular interplay between tumor cells and perirenal adipocytes in order to design novel therapeutic approaches in oncologic patients.

## 7. PRAT-Targeted Therapeutic Perspectives in Cancer

Preclinical and clinical studies have recently opened new directions by targeting PRAT-derived adipokines or PRAT-adapted morphology and metabolism in cancer therapy [19,21]. One major area of research is related to the modulation of the uptake of tumor cells of adipocyte-derived lipids, which are useful for their growth and metastasis. In this regard, the pro-tumoral activity of G protein-coupled receptor 1 (GPR1) and CMKLR1 chemerin receptors has been recently demonstrated to induce FAAs uptake and the clear cell morphologic features in ccRCC, by modulation of tumor cells’ SREBP1c and CD36 expression [24,156]. The Chemerin-CMKLR1 axis is also involved in the recruitment of local macrophages, contributing to tumor microenvironment immune suppression [157]. Consequently, targeting PRAT-derived chemerin and its receptors may represent a new direction in oncotherapy. A recent study of Wang et al. has shown that α-NETA, a CMKLR1 inhibitor, suppresses renal tumor cell development and lipid storage in ccRCC-derived xenograft models (XP296) and in the UOK101 cell line [21]. Therefore, a comprehensive understanding of the mechanism of PRAT-derived chemerin modulation of the lipid metabolism in cancer and tumor-associated macrophage activity may offer new perspectives for RCC therapy.

PRAT-increased lipolysis is also promoted by HIF overexpression in the hypoxic microenvironment [107,158]. Although HIFs are associated with increased drug resistance, the first generation of HIF-2α inhibitors, such as TC-S 7009 or PT-2399, were tested in preclinical studies in ccRCC xenografts and in human ccRCC cell lines [23,159]. However, a decrease of tumor size and downregulation of the HIF-2α specific genes PAI-1, CCND1, EPO, and GLUT-1 have been reported in ccRCC xenografts treated with PT-2385 (aka MK-3795), a new HIF-2α inhibitor [160]. Recently, PT-2385 has been tested in phase I clinical trials for RCC therapy [161], but common adverse reactions have been related to its antitumoral effect, such as anemia, edema, and fatigue, as well as HIF-2α gene mutation in some cases [161]. Moreover, HIF-2α and CDK4/6 inhibition may have a synergic antiproliferative effect on tumor cells, most probably by modulation of the tumor cells’ transition to the G1 phase [162]. In this respect, administration of abemaciclib, a CDK4/6 inhibitor, is currently being investigated in a phase 1 trial (NCT04627064), as monotherapy or in combination with belzutifan [158]. Therefore, the improved antitumoral response and PRAT adipocyte lipolysis decrease achieved through HIF expression targeting may be an additional therapeutic option in RCC.

On the other hand, the PRAT ‘browning’ process is promoted by tumor cells, which may act as an additional factor for cancer cell progression. In this regard, several researchers have shown that the PRAT ‘browning’ process may be induced by TKIs, such as axitinib or sunitinib [163,164], through brown adipocyte marker overexpression (e.g., UCP1, Dio2, and Pgc1α), added to genes that support lipolysis (*Atgl* and *Hsl*), in white adipocytes [19]. Additionally, a significant reduction of tumor mass has been identified in xenograft tumor-bearing mice treated with sunitinib and H89 or sunitinib associated with KT5720 tyrosine kinase inhibitor [19]. The renal cancer cells are able to release parathyroid-hormone-related protein (PTHrP), supporting this brown-to-white transformation via PKA activation, highlighting PRAT’s complex role in cancer risk [19]. All these findings indicate that combined therapy, including TKIs and thermogenesis inhibitors, may be added to the onco-therapeutic arsenal in RCC patients [19]. Moreover, IL-6 stimulates delipidation in adipocytes through JAK/STAT pathway activation [165]. Additionally, cancer cell-derived extracellular vesicles contain IL-6, which promotes HSL phosphorylation and increased lipolysis [165]. As a consequence, prevention of IL-6 activity by specific neutralizing antibodies or STAT3 inhibitors may represent another potential research area of new therapeutic agents to prevent lipolysis, brown adipocyte conversion, and cancer-associated cachexia. Furthermore, pharmacological inhibition of white-to-brown adipocyte transformation in PRAT may enhance the antitumoral role of sunitinib [19].

Several studies have revealed the role of PRAT-derived adiponectin in cancer [11,54,166]. A study has shown that adiponectin may induce an anti-angiogenic and metastatic inhibitory capacity in RCC cells by AMPK pathway activation and decreased secretion of MMP2, MMP-9, and VEGF [167]. In support of these findings, a recent study revealed that adiponectin decreases RCC cells’ motility through p-GSK-3β/β-catenin inhibition [11]. Moreover, according to the same study, adiponectin may increase the renal cancer cells’ sensitivity to sunitinib by GSK-3β/β-catenin pathway downregulation [11]. As a consequence, AdipoR1 may be considered a new predictor of TKIs’ response and a potential therapeutic candidate for overcoming RCC chemotherapy resistance. However, supplementary clinical studies are necessary to explore the anti-angiogenic role of co-administration of adiponectin and sunitinib in RCCs. Last but not least, a new AdipoR1 agonist has been found to display a potential antitumoral role in pancreatic and breast cancers [25,168]. Consequently, adiponectin and AdipoR1 agonists have been proposed as novel therapeutic tools in mRCC patients with TKI resistance.

Leptin treatments are not currently applied in cancer, since some data suggests a dual role of leptin in carcinogenesis [27]. However, leptin antagonists may be exploited in novel antitumoral therapeutic strategies to overcome chemotherapeutic resistance [169]. Currently, several leptin antagonists, such as LDFI, leptin muteins (SMLA and SHLA), leptin peptide receptor antagonists (LPrA), D-ser, and Allo-aca, have been tested in association with conventional therapy, leading to variable results [169]. Among these leptin antagonists, only LPrA2 has been certified as effective in the decrease of leptin-induced effects in breast cancer patients with multidrug resistance to sunitinib, doxorubicin, cisplatin, and paclitaxel [170,171]. Although targeting PRAT-derived leptin has not been studied, leptin agonists may constitute a new therapeutic adjuvant, since they do not display toxicity and may control the balance between the expended and intake energy.

Additionally, leptin may act as a modulator of innate and adaptive immune responses in cancer. In this regard, leptin may induce B and T cell proliferation and may increase NK cells’ cytotoxicity, supporting the immune response in breast cancer [172,173]. Moreover, leptin downregulates Treg cell differentiation, leading to a poor prognosis in different types of cancers [27]. Consequently, leptin may be a potential therapeutic candidate for improvement of immunotherapy responses.

PRAT beige adipocytes release an increased level of lactate in the tumor microenvironment, supporting the cancer cells’ increased metabolism. Consequently, targeting lactate catabolism may open a potential therapeutic window in oncologic patients. In this regard, inhibition of monocarboxylate transporter 1 (MCT1) and MCT4 with 7ACC1 small molecule has been shown to decrease RCC tumor cells’ aggressiveness [19]. In support of these findings, analogous data have been reported in a study on 786-O and Caki-1 RCC cell lines by Miranda-Gonçalves et al. [26]. Alpha-cyano-4-hydroxycinnamate, a lactate transporter inhibitor, may also promote SIRT1 overexpression and decreased N-cadherin expression of cancer cells, leading to reduced tumor cell migration and reduced cancer cell aggressiveness in RCC [26]. Based on these preliminary data, MCT inhibitors or other agents that improve SIRT1 activity, e.g., resveratrol, may be added to current therapeutic tools in RCC patients.

Last but not least, PPARs are potential therapeutic targeted PPARs in oncologic patients. Nevertheless, very few studies have focused on determining PPARs’ therapeutic potential in renal cancer and other malignancies related to PRAT [174]. In this respect, only Abu Aboud et al. reported that GW6471, a PPARα agonist, has an anti-tumor activity induced by inhibiting cyclin D1, CDK4, and c-Myc, leading to apoptosis induction in cancer cells [22].

Considering these cumulative data, PRAT activity modulation may be a promising alternative antitumor therapeutic strategy in oncologic patients, which may inhibit tumor development and ameliorate multidrug resistance.

## 8. Conclusions

PRAT is a unique visceral fat deposit that plays a significant role in the human body’s homeostasis. Recently, perirenal adipocyte dysfunction has been associated with renal and cardiovascular diseases, diabetes mellitus, and cancer, in obese patients.

Cancer cells are able to induce a morphologic and metabolic adaptation of perirenal adipocytes to support their excessive needs. The multiple effects of PRAT in different stages of tumor cell growth and metastasis highlight its potential as an additional therapeutic target in cancer. However, due to their secretion of adipokines, a dual role has been attributed to PRAT adipocytes in different cancer types. The duality of PRAT-cancer cells’ relationship demonstrates the specific anatomical, morphological, and molecular characteristics of this visceral fat type.

The controversial PRAT role in cancer progression is most probably related to tumor histopathological spectrum diversity, but also to different study designs, either in experimental models, cell lines cultures, or clinical studies. Moreover, the data discrepancies may be attributed to the molecular complexity of the tumor microenvironment and to the partially deciphered complex interaction between PRAT and tumor cells.

Due to limited data and sometimes contradictory results, a deeper understanding of the molecular interrelationship between cancer cells and PRAT may provide the foundation of future personalized therapy development, favoring the establishment of an anti-tumor microenvironment and leading to improved oncologic patient prognosis.
